# Habitat Filtering Shapes Root Endophytic Microbiome Assembly and Its Association with Fruit Quality in *Lycium ruthenicum* from the Tarim Basin

**DOI:** 10.3390/plants15060979

**Published:** 2026-03-22

**Authors:** Aihua Liang, Fengjiao Wang, Tianyi Liu, Yuting Liao, Zixin Mu

**Affiliations:** 1 College of Life Science & Technology, Tarim University, Alar 843300, China; aiai_1025@163.com (A.L.); 120220045@taru.edu.cn (F.W.); 18382772150@163.com (T.L.); l7011220213@163.com (Y.L.); 2 State Key Laboratory Incubation Base for Conservation and Utilization of Bio-Resource in Tarim Basin, Tarim University, Alar 843300, China

**Keywords:** *Lycium ruthenicum*, Tarim Basin, root endophytes, microbial diversity, community structure, co-occurrence network, environmental factors, fruit quality

## Abstract

*Lycium ruthenicum* is a typical desert halophyte with strong stress resistance and high medicinal value in the Tarim Basin. Root endophytic microbes play critical roles in host adaptation, nutrient cycling, and secondary metabolite accumulation. To clarify the diversity patterns of root endophytic bacteria and fungi and their relationships with environmental factors and fruit quality, high-throughput sequencing was used to analyze microbial community characteristics of *Lycium ruthenicum* collected from different habitats in the Tarim Basin. The results showed that rarefaction curves of alpha diversity indices (Chao1, Shannon, Pielou_e) tended to be saturated, indicating sufficient sequencing depth. Principal coordinate analysis (PCoA) revealed significant habitat-driven differentiation in both bacterial and fungal community structures. Community composition analysis showed that the relative abundance of dominant taxa at the phylum and genus levels differed significantly among sampling sites. Co-occurrence network analysis indicated that bacterial and fungal networks exhibited high modularity and were dominated by positive synergistic interactions, with *Pseudomonas*, *Bacillus*, *Sphingomonas*, *Alternaria*, and *Fusarium* as key hub genera. Moreover, root endophytic communities were significantly correlated with climatic variables, soil physicochemical properties, and fruit quality traits, including anthocyanin (AC), proanthocyanidin (PA), total flavonoids (TF), and total polyphenols (TP). Several keystone microbial genera were closely associated with the accumulation of functional metabolites in fruits. This study reveals the biogeographic distribution and co-occurrence characteristics of root endophytes in *Lycium ruthenicum* and provides a theoretical basis for understanding microbe–host–environment interactions and the quality improvement of desert medicinal plants.

## 1. Introduction

Endophytes are ubiquitous inhabitants of the specialized microenvironments within plant tissues, having undergone long-term co-evolution with their hosts to form stable mutualistic symbiotic relationships. Host plants provide endophytes with photosynthates and mineral nutrients for growth and reproduction, while endophytes play pivotal roles in regulating host growth, development, and adaptive evolution [1]. Among them, root endophytes—fungi and bacteria that colonize plant root tissues without causing visible pathogenic symptoms—are recognized as key contributors to host plant performance [2]. They facilitate host acquisition of nutrients such as phosphorus and nitrogen, enhance tolerance to abiotic stresses, including drought, salinity, and heavy metal toxicity [3], and directly or indirectly modulate plant secondary metabolic and defense pathways [4]. Thus, the assembly and composition of root endophytic communities are critical determinants of plant growth, health, and the quality of harvested products [5]. Notably, root endophytic microbial communities are not static; their structure is dynamically shaped by the complex interplay of host genotype, soil properties, and local habitat conditions (e.g., pH, moisture, salinity, and vegetation history) [6]. This habitat-specific microbiome assembly results in distinct endophytic consortia in plants across different geographical or ecological niches, which in turn exert differential effects on host physiological processes [7].

A growing body of research has confirmed that abiotic factors in different geographical regions drive the differentiation of soil microbial communities, which further regulate the rhizosphere microenvironment and secondary metabolite accumulation in medicinal plants [8]. This microbial-mediated regulation not only enhances herbal quality but also underpins the formation of Daodi medicinal materials—a core concept in traditional Chinese medicine (TCM) that links superior herbal quality to specific geographic origins [9]. Biogeographical differentiation of soil microbes is therefore essential for understanding the molecular and ecological mechanisms of Daodi herb formation. To date, endophytic communities in nearly 100 medicinal plant species have been shown to exhibit significant geographical variation, particularly between Daodi and non-Daodi habitats [9]. Daodi medicinal plants typically harbor higher endophytic biodiversity and more endemic endophytic taxa [10], a feature shaped by adaptive responses to local environmental stress: moderate stress induces the synthesis of specialized secondary metabolites, leading stress-tolerant cultivars to produce richer and more abundant bioactive compounds [11]. Endophytes, including both fungi and bacteria, mediate the accumulation of secondary metabolites in a geographically and tissue-specific manner [12], and their synergy with host plants forms unique TCM microecosystems that generate distinct microecological effects, ultimately leading to regional differences in herbal quality [13]. Elucidating the biogeographical patterns and regulatory mechanisms of soil and endophytic microbes is thus critical for deciphering Daodi herb formation and guiding the targeted cultivation of high-quality medicinal plants [5].

*Lycium ruthenicum* Murray (black goji) is a perennial halophytic shrub endemic to the arid and semi-arid regions of northwest China, with extensive distribution across the Tarim Basin [14]. It is highly valued for its exceptional resilience to harsh saline-alkali environments and for its fruits, which are rich in anthocyanins, polysaccharides, and other bioactive compounds with prominent antioxidant and medicinal properties [15]. Escalating commercial demand for its health-promoting fruits has focused research attention on the factors governing fruit yield and phytochemical quality, with traditional studies centering on abiotic stressors (e.g., soil salinity, drought, and mineral nutrition) and plant genetic factors. However, the root endophytic microbiome—a key biological component modulating plant fitness and metabolism in natural ecosystems—remains under-explored in *Lycium ruthenicum*. In the Tarim Basin, *Lycium ruthenicum* populations are distributed across a mosaic of heterogeneous habitats, from severely saline deserts to riparian zones with divergent soil physicochemical and microclimatic conditions, presenting a natural experimental system to investigate how habitat filtering drives variation in root endophytic microbiomes. A critical knowledge gap persists: while the functional potential of endophytes has been well established in model plant systems, empirical evidence linking natural variation in *Lycium ruthenicum* root endophytic communities to the quantitative and qualitative traits of its economically vital fruits is scarce. It remains unclear whether habitat-driven divergence in the root microbiome correlates with measurable differences in fruit biomass, anthocyanin content, sugar–acid balance, or antioxidant capacity. Resolving this gap is essential for moving beyond correlative analyses to understanding the potential microbial contributions to fruit quality in this ecologically and economically important halophyte [16,17].

In this study, high-throughput sequencing was employed to characterize the alpha diversity, beta diversity, community composition, and co-occurrence patterns of root endophytic bacteria and fungi in *Lycium ruthenicum* across distinct sampling sites in the Tarim Basin. We further analyzed the correlations between root endophytic communities and climatic factors and soil physicochemical properties, as well as key fruit quality indicators. The specific objectives were to: (1) reveal the variation in root endophytic diversity and community structure driven by habitat heterogeneity; (2) explore the intra-kingdom co-occurrence relationships within bacterial and fungal endophytic communities; and (3) clarify the potential linkage between root endophytic community characteristics and fruit quality formation. This study provides novel insights into the microbe-mediated quality formation of desert medicinal plants and offers a scientific basis for the development and utilization of beneficial endophytic microbial resources to improve the quality of *Lycium ruthenicum* fruits [18].

## 2. Results

### 2.1. Analysis of Alpha Diversity

**Figure 1:**
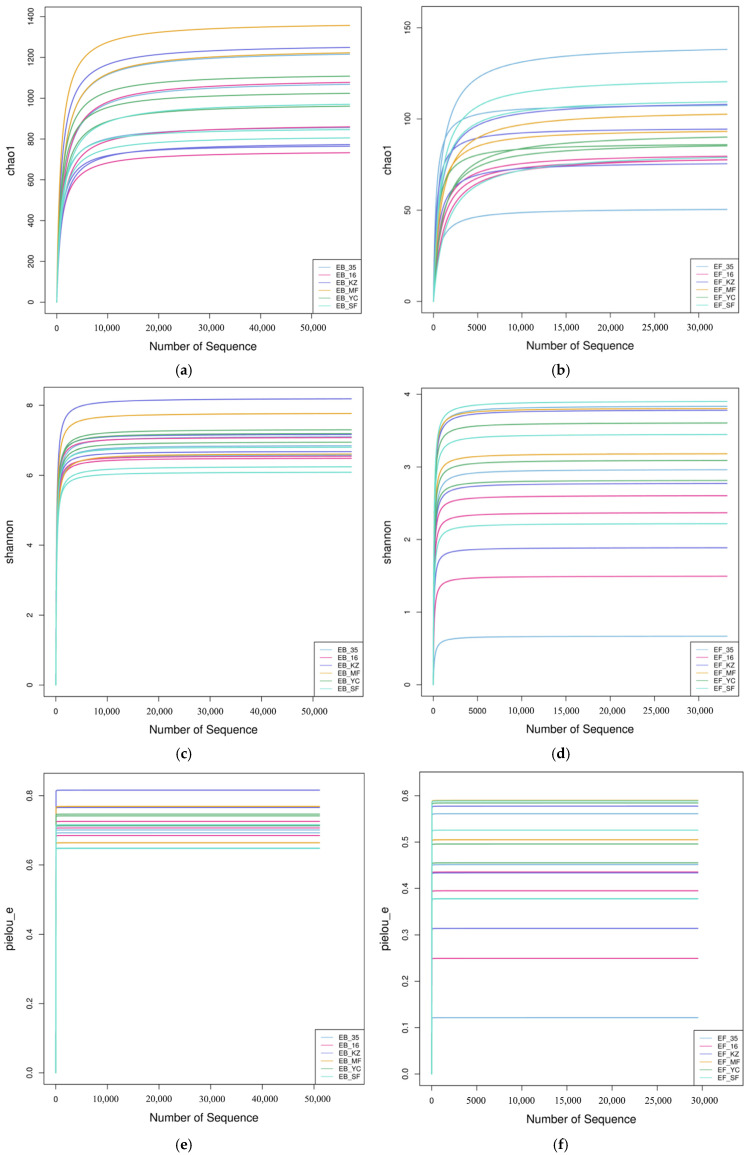
Rarefaction curves of alpha diversity indices for root endophytic bacteria and fungi of *Lycium ruthenicum* from the Tarim Basin. (**a**,**c**,**e**) Rarefaction curves of Chao1, Shannon, and Pielou evenness indices for endophytic bacteria; (**b**,**d**,**f**) Rarefaction curves of Chao1, Shannon, and Pielou evenness indices for endophytic fungi. The x-axis represents the number of sequencing reads, and the y-axis represents the corresponding alpha diversity index. Different colored lines represent different sampling groups. EB: endogenous bacteria, EF: endogenous fungi, 35: Yuli County, MF: Minfeng County, YC: Yecheng County, KZ: Aketao County, SF: Shufu County, 16: Awati County.

Alpha diversity, also known as within-habitat diversity, can indicate the diversity within a specific area or ecosystem, reflecting the number, abundance, and evenness of species in a sample. Rarefaction curves based on alpha diversity indices, including Chao1, Shannon, and Pielou evenness, were constructed to evaluate the sequencing depth and species richness of root endophytic bacterial and fungal communities in *Lycium ruthenicum* collected from different regions of the Tarim Basin. For bacterial communities, the rarefaction curves of Chao1, Shannon, and Pielou evenness indices all rose rapidly at the initial sequencing stage and gradually approached a smooth plateau with the increase in sequencing quantity, without obvious upward trends (Figure 1). Similarly, the rarefaction curves of fungal communities exhibited the same variation tendency, and all curves tended to be saturated and flattened gradually. These results indicated that the current sequencing depth was sufficient and reasonable to capture the majority of species diversity, and the sequencing data volume could fully reflect the real alpha diversity characteristics of endophytic bacterial and fungal communities in all sampling groups. In addition, obvious separations among different sampling groups were observed in all rarefaction curves, suggesting that there were distinct differences in alpha diversity patterns of root endophytic microbes among different habitats of the Tarim Basin.

### 2.2. Beta Diversity Analysis

**Figure 2:**
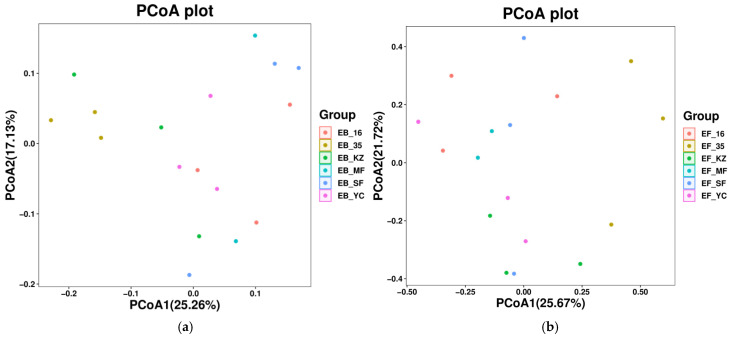
Principal coordinate analysis (PCoA) of root endophytic bacterial (**a**) and fungal (**b**) communities of *Lycium ruthenicum*. PCoA was performed based on Bray–Curtis distance. For bacteria, PCoA1 and PCoA2 explained 25.26% and 17.13% of the total variation, respectively. For fungi, PCoA1 and PCoA2 explained 25.67% and 21.72% of the total variation, respectively. Different colors indicate different sampling habitats. EB: endogenous bacteria, EF: endogenous fungi, 35: Yuli County, MF: Minfeng County, YC: Yecheng County, KZ: Aketao County, SF: Shufu County, 16: Awati County.

Principal coordinate analysis (PCoA) based on Bray–Curtis distance was performed to reveal the beta-diversity patterns of root endophytic bacterial and fungal communities in *Lycium ruthenicum* from different habitats of the Tarim Basin. For endophytic bacteria (Figure 2a), the first two principal axes explained 25.26% and 17.13% of the total variation, respectively. The sampling groups were clearly separated along the PCoA1 and PCoA2 axes, with obvious spatial segregation and limited overlap among different groups, indicating that the community structure of root endophytic bacteria differed significantly among distinct geographical habitats.

For endophytic fungi, PCoA1 and PCoA2 accounted for 25.67% and 21.72% of the total variation, respectively (Figure 2b). The distribution of fungal communities also exhibited distinct clustering patterns, and samples from different groups were dispersed separately in the ordination space. The obvious separation among groups suggested that the endophytic fungal community structure was also shaped by habitat heterogeneity in the Tarim Basin. Overall, both endophytic bacterial and fungal communities showed significant spatial differentiation among sampling sites, and the geographical habitat was a key driving factor for the beta-diversity variation in root endophytic microbes in *Lycium ruthenicum*.

### 2.3. Analysis of Endophyte Community Structures of Samples from Different Regions

**Figure 3:**
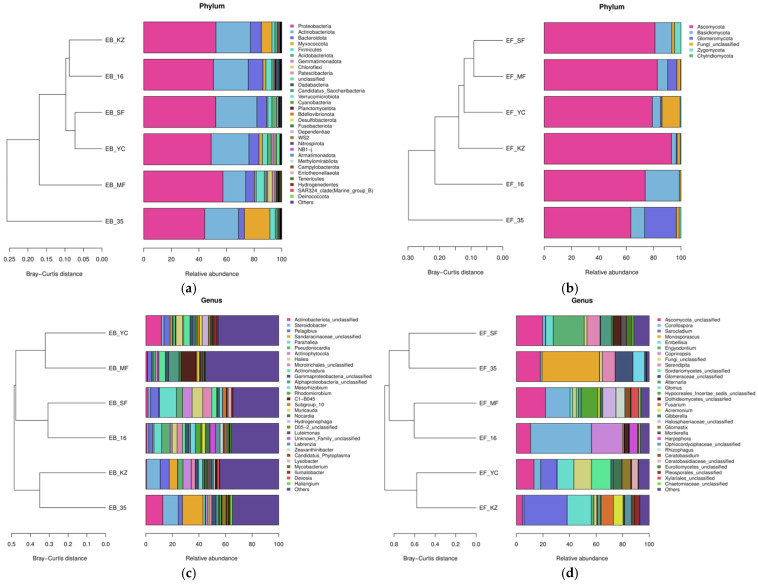
Community composition and hierarchical clustering of root endophytic microbes of *Lycium ruthenicum* at the phylum and genus levels. (**a**) Bacterial community composition at the phylum level; (**b**) fungal community composition at the phylum level; (**c**) bacterial community composition at the genus level; (**d**) fungal community composition at the genus level. Bar plots show the relative abundance of dominant taxa, and clustering trees were constructed based on Bray–Curtis distance. EB: endogenous bacteria, EF: endogenous fungi, 35: Yuli County, MF: Minfeng County, YC: Yecheng County, KZ: Aketao County, SF: Shufu County, 16: Awati County.

To characterize *Lycium ruthenicum* root endophytic communities across six Tarim Basin sites (16, KZ, MF, SF, YC, 35), taxonomic composition and variation were analyzed at phylum and genus levels. At the phylum level (Figure 3a,b), endophytic bacteria were dominated by *Proteobacteria* (>40% relative abundance across regions), with minor contributions from *Actinobacteriota* and *Bacteroidota*; Bray–Curtis clustering grouped sites 16/YC/KZ/SF separately from MF/35. Endophytic fungi were dominated by *Ascomycota* (>60% relative abundance across regions), with rare phyla (*Basidiomycota*, *Glomeromycota*) accounting for small proportions; site clustering reflected limited phylum-level variation.

At the genus level (Figure 3c,d), bacterial communities showed distinct region-specific variation: *Steroidobacter* dominated region KZ, while *Parahaliea* were abundant at SF; Bray–Curtis clustering separated 35/KZ from other regions. Fungal communities exhibited genus-level heterogeneity: *Corollospora*, *Sarocladium*, and *Monosporacus* were prevalent at 16, KZ and 35 region, respectively; regions 16/MF clustered closely via shared taxa.

These data indicate that *Lycium ruthenicum* root endophytic communities in the Tarim Basin maintain consistent phylum-level dominance but exhibit region-specific variation at the genus level, driven by habitat-associated environmental factors.

### 2.4. Co-Occurrence Network Analysis Results

**Figure 4:**
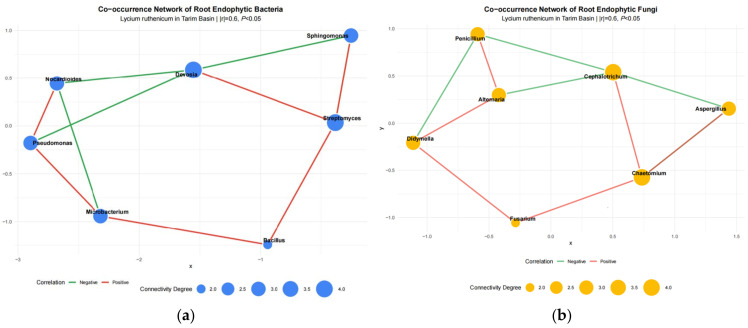
Co-occurrence networks of root endophytic bacterial (**a**) and fungal (**b**) communities of *Lycium ruthenicum*. Networks were constructed based on Pearson correlation (|r| ≥ 0.6, *p* < 0.05). Blue and yellow nodes represent bacterial and fungal genera, respectively. Node size is proportional to connectivity degree. Red solid edges and green dashed edges indicate significant positive and negative correlations, respectively. Networks were visualized using the ForceAtlas2 layout algorithm.

Co-occurrence network analysis was performed to explore the intra-kingdom interactions among root endophytic bacteria and fungi. As shown in Figure 4, both bacterial and fungal networks exhibited clear topological structure with distinct modularity. For the bacterial network (Figure 4a), *Streptomyces* and *Devosia* were identified as the hub nodes with larger node sizes and higher connectivity degrees, suggesting their critical roles in maintaining the stability of the bacterial community. Most edges were positive correlations, indicating that synergistic interactions were dominant among root endophytic bacteria. For the fungal network (Figure 4b), *Cephalotrichum* and *Chaetomium* served as the core keystone genera with relatively high connectivity. Similar to bacteria, positive correlations also accounted for the majority of interactions in the fungal community, reflecting intense synergistic coexistence among fungal taxa.

Overall, both bacterial and fungal co-occurrence networks exhibited clear modularity and were dominated by positive interactions, indicating that cooperative symbiosis plays a crucial role in the community assembly of root endophytes in *Lycium ruthenicum* across the Tarim Basin.

### 2.5. Pearson Correlation Analysis of Root Endophytes and Environmental Factors

**Figure 5:**
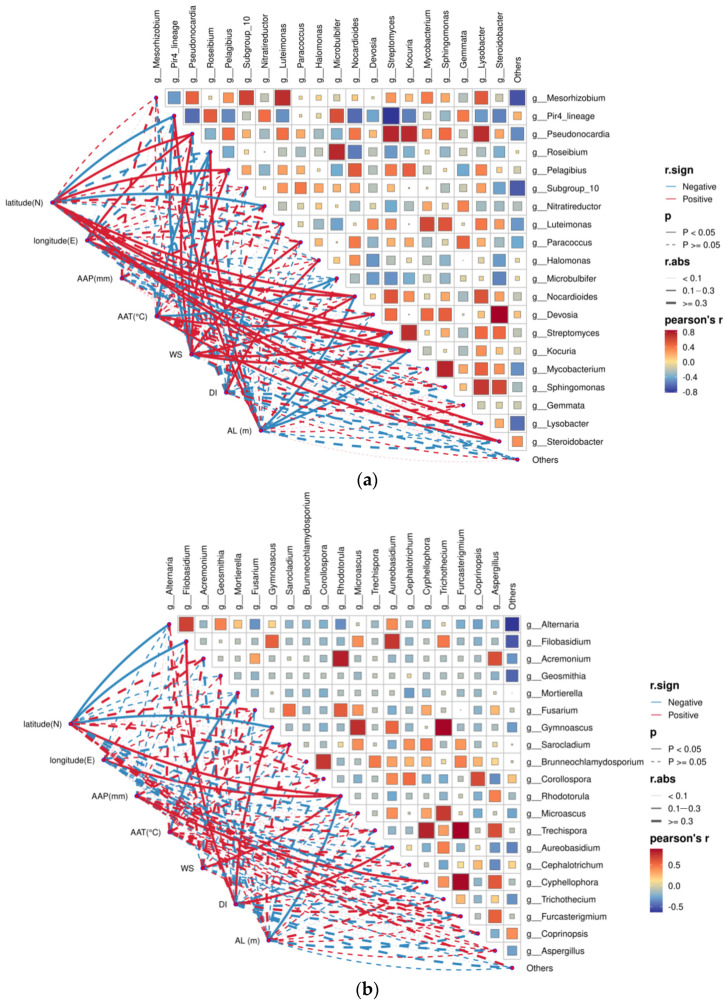
Pearson correlation analysis between dominant endophytic microbial genera and climatic factors. (**a**) Correlations between bacterial genera and climatic variables; (**b**) correlations between fungal genera and climatic variables. The heatmap shows Pearson’s correlation coefficient (r). Red and blue solid lines indicate positive and negative correlations, respectively. The dashed lines indicate that there is no significant correlation between the two indicators. AAP: annual average precipitation, AAT: annual average temperature, WS: wind speed, DI: drought index, AL: altitude. Significance levels: *p* < 0.05; *p* < 0.01.

Pearson correlation analysis was conducted to explore the links between climatic and geographical factors and the composition of bacterial and fungal communities at the genus level. For the bacterial community, latitude and wind speed (WS) were both significantly positively correlated with *Streptomyces*, *Nocardioides*, *Kocuria*, *Pseudonocardia*, and *Pelagibius* (Pearson’s r > 0.4) (Figure 5a). Latitude was significantly negatively correlated with *Pir4_lineage* and *Nitratireductor*. Wind speed was significantly negatively correlated with *Pir4_lineage* and *Roseibium*, whereas altitude (AL) was positively correlated with these two genera. Meanwhile, AL was significantly negatively correlated with *Streptomyces*, *Nocardioides*, and *Kocuria*. Longitude was significantly positively correlated with *Streptomyces*, *Nocardioides*, and *Pseudonocardia*. Annual average temperature (AAT) was negatively correlated with *Roseibium* and *Microbulbifer*, but significantly positively correlated with *Devosia* and *Steroidobacter*. Aridity index (DI) was positively correlated with *Halomonas* and negatively correlated with *Luteimonas*. Annual average precipitation (AAP) showed no significant correlation with any bacterial genus.

For the fungal community, latitude was significantly negatively correlated with genera including *Alternaria*, *Filobasidium*, and *Gymnoascus* (Figure 5b). *Rhodotorula* was significantly positively correlated with latitude, longitude, and WS, but significantly negatively correlated with AL, making it the fungal genus with the most complex associations with environmental factors. DI was significantly positively correlated with *Filobasidium*, *Gymnoascus*, and *Aureobasidium*.

**Figure 6:**
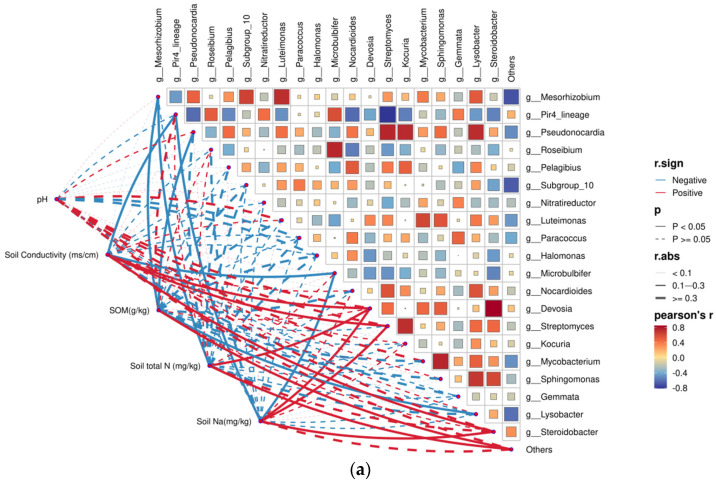

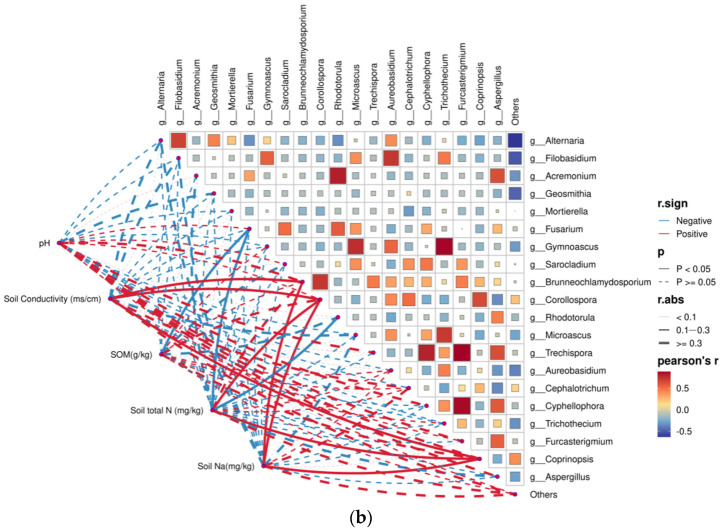
Pearson correlation analysis between dominant endophytic microbial genera and soil physicochemical properties. (**a**) Correlations between bacterial genera and soil factors; (**b**) correlations between fungal genera and soil factors. The color gradient indicates the magnitude of Pearson’s r. Red and blue solid lines represent positive and negative correlations, respectively. The dashed lines indicate that there is no significant correlation between the two indicators. Significance levels: *p* < 0.05; *p* < 0.01.

To characterize links between *Lycium ruthenicum* root endophytic communities and soil properties (pH, conductivity, SOM, total N, and Na) in the Tarim Basin, Pearson’s correlation analysis was conducted for endophytic bacteria (Figure 6a) and fungi (Figure 6b). Results revealed extensive and significant correlations between the bacterial community and soil environmental factors (*p* < 0.05). Specifically, genera including *Devosia*, *Steroidobacter*, and *Streptomyces* showed significantly positive correlations with soil EC, Na content, and TN content (Pearson’s r > 0.4), indicating that these taxa prefer saline and eutrophic soil habitats. *Mesorhizobium* exhibited significantly negative correlations with SOM and TN, suggesting that this genus is more adapted to nutrient-poor soil environments. *Pir4_lineage* and *Microbulbifer* were significantly negatively correlated with soil Na content and EC, demonstrating distinct salt sensitivity and poor tolerance to high-salt conditions. In addition, *Pseudonocardia* and *Lysobacter* were also significantly negatively correlated with SOM, while soil pH showed no significant correlation with any detected bacterial genus.

Compared with the bacterial community, fewer fungal genera were significantly correlated with soil physicochemical properties. In detail, *Corollospora* and *Coprinopsis* were significantly positively correlated with soil EC, Na content, and TN, while *Brunneochlamydosporium* was significantly positively correlated with EC and Na content. *Fusarium* was significantly negatively correlated with SOM and TN, and *Rhodotorula* was significantly negatively correlated with TN. Overall, taxa including *Devosia*, *Corollospora*, and *Coprinopsis* displayed the closest associations with soil physicochemical properties.

### 2.6. Pearson Correlation Analysis of Root Endophytes and Fruit Quality Traits

**Figure 7:**
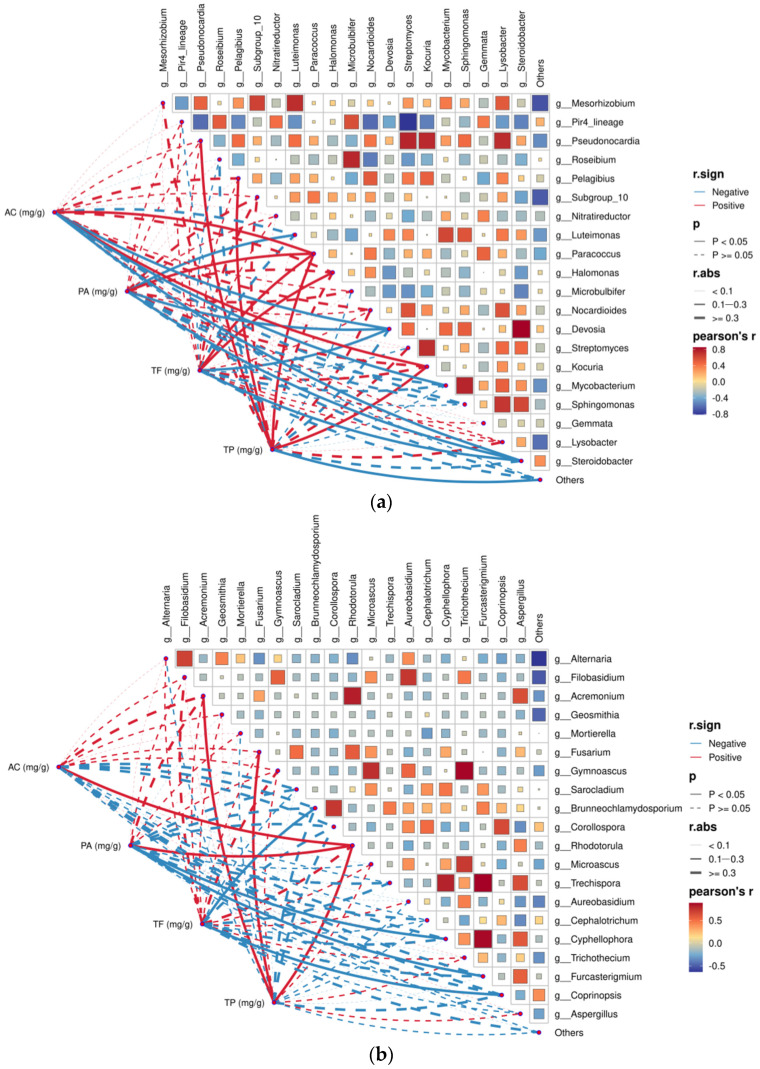
Pearson correlation analysis between dominant endophytic microbial genera and fruit quality traits. (**a**) Correlations between bacterial genera and fruit quality indexes; (**b**) correlations between fungal genera and fruit quality indexes. The color gradient indicates the magnitude of Pearson’s r. Red and blue solid lines represent positive and negative correlations, respectively. The dashed lines indicate that there is no significant correlation between the two indicators. AC: anthocyanin; PA: proanthocyanidin; TF: total flavonoids; TP: total polyphenols. Significance levels: *p* < 0.05; *p* < 0.01.

To clarify links between root endophytic microorganisms and fruit functional components of *Lycium ruthenicum* in the Tarim Basin, Pearson’s correlation analysis was conducted for root endophytic bacteria (Figure 7a) and fungi (Figure 7b) against fruit anthocyanin (AC), proanthocyanidin (PA), total flavonoids (TF), and total polyphenols (TP). Within the bacterial community, *Paracoccus* was significantly positively correlated with fruit AC, PA, TF, and TP. *Pelagibius*, *Pseudonocardia*, and *Kocuria* were all significantly positively correlated with TP (Pearson’s r > 0.4). In contrast, *Devosia* and *Steroidobacter* were significantly negatively correlated with AC, PA, and TF. Based on these correlation results, it is speculated that *Paracoccus* positively regulates wolfberry fruit quality, while *Devosia* and *Steroidobacter* negatively regulate fruit quality.

Within the fungal community, *Rhodotorula* was significantly positively correlated with AC, PA, and TP, representing the fungal genus with the strongest correlation with fruit quality traits. *Acremonium* and *Fusarium* were significantly positively correlated with TP. *Gyphellophora* and *Furcasterigmium* were significantly negatively correlated with PA, while *Coprinopsis* and *Brunneochlamydosporium* were significantly negatively correlated with TF.

## 3. Discussion

The rarefaction curves reaching a plateau indicate that the sequencing depth is sufficient to capture most of the bacterial and fungal species in all samples, and the differences in diversity indexes (Chao1, Shannon, and Pielou) among samples are real and not caused by insufficient sequencing depth. We explain the lower diversity indexes of the blue-curve samples (fungi, Figure 1b,d,f) as follows: (1) Environmental factors: The sampling points corresponding to these blue-curve samples have specific soil properties (e.g., higher soil Na content or lower SOM content, as shown in Table A2), which may inhibit the growth and colonization of some fungal species, leading to lower diversity; (2) Plant-microbe interaction: The plants in these sampling points may have stronger selective screening on endophytic fungi, preferentially colonizing specific fungal taxa and excluding others, resulting in reduced fungal diversity; (3) Microbial competition: The dominant fungal taxa in these samples may have strong competitive advantages, occupying most of the ecological niches, thereby suppressing the growth of other fungal species and reducing community evenness (reflected in the lower Pielou index).

This study provides consistent evidence that the root endophytic microbiome of *Lycium ruthenicum* is not a random assembly but is strongly shaped by habitat filtering across heterogeneous environments in the Tarim Basin. The significant divergence in endophytic community composition among sampling sites, as revealed by PCoA and hierarchical clustering, fully supports the ecological paradigm that environmental heterogeneity acts as a key filter driving microbial biogeographic patterns [14]. The relatively high abundance of halotolerant and drought-resistant microbial taxa in harsh habitats further implies an adaptive recruitment strategy, in which the host plant preferentially associates with endophytes capable of enhancing host tolerance to local abiotic stresses, such as drought, salinity and nutrient deficiency [3,19,20,21]. Such habitat-specific microbiome assembly provides a foundational basis for divergent functional effects on host physiology and metabolic output [7].

Notably, our results demonstrate significant correlations between root endophytic community characteristics and multiple key fruit quality traits, including AC, PA, TF, and TP. Specifically, several dominant bacterial genera (e.g., *Paracoccus*, *Pelagibius*, *Pseudonocardia* and *Kocuria*) and core fungal taxa (e.g., *Phodotorula*, *Acremonium*, and *Fusarium*) were strongly associated with elevated levels of bioactive metabolites. These genera are widely recognized as plant growth-promoting endophytes that can enhance nutrient acquisition, induce systemic stress tolerance, and regulate host secondary metabolism [2,22,23]. Based on our combined network and correlation analyses, we propose a conceptual framework whereby habitat-modulated endophytes may contribute to fruit quality through three complementary pathways: (1) improving host uptake and translocation of essential nutrients, thereby allocating more resources to fruit development and metabolite accumulation [16]; (2) priming host stress-responsive pathways, which indirectly upregulate the biosynthesis of phenolics, flavonoids, and anthocyanins as adaptive antioxidants [11]; and (3) producing phytohormones or signaling molecules that directly modulate metabolic fluxes in reproductive tissues [24,25].

Co-occurrence network analysis further revealed that both bacterial and fungal endophytic communities exhibited high modularity and were dominated by positive interactions, indicating intensive synergistic cooperation among microbial taxa. Hub genera with high connectivity, such as *Pseudomonas* and *Bacillus* in bacteria, and *Alternaria* and *Fusarium* in fungi, may play keystone roles in maintaining community stability and functional coherence [15]. These structural features likely enhance the overall resilience of the endophytic microbiome under extreme desert conditions, which in turn supports host adaptation and consistent fruit quality formation [26].

By linking natural variation in the root endophytic microbiome directly to economically important fruit quality traits, our findings fill a critical knowledge gap for *Lycium ruthenicum*. These results highlight that the ecological performance and phytochemical characteristics of *Lycium ruthenicum* should be interpreted as emergent properties of the plant-microbe holobiont, shaped jointly by host genetics and habitat-specific environmental filtering [7,27,28]. This perspective advances the understanding of Daodi medicinal herb formation from a microbial ecological viewpoint, emphasizing that endophytic microbiota are indispensable components underlying the quality and stability of medicinal plants [9,13].

Nevertheless, several limitations should be acknowledged. First, this study is based on observational and correlative evidence; thus, causal relationships between specific endophytes and fruit quality traits remain to be validated. Targeted inoculation experiments using gnotobiotic or sterile systems will be necessary to confirm the functional roles of keystone taxa [4,24]. Second, the molecular mechanisms and metabolic pathways by which endophytes regulate fruit quality remain unresolved. Further studies combining metagenomics, metabolomics, and transcriptomics are needed to decode the functional potential of endophytic communities and identify key genes or enzymes involved in modulating secondary metabolism [5]. Overall, this study enhances our understanding of habitat-driven endophytic microbiome assembly and its association with fruit quality in desert halophytes and provides a scientific basis for the future utilization of beneficial endophytes to improve the yield and quality of *Lycium ruthenicum* [13,18].

## 4. Materials and Methods

### 4.1. Sampling Sites

**Figure 8:**
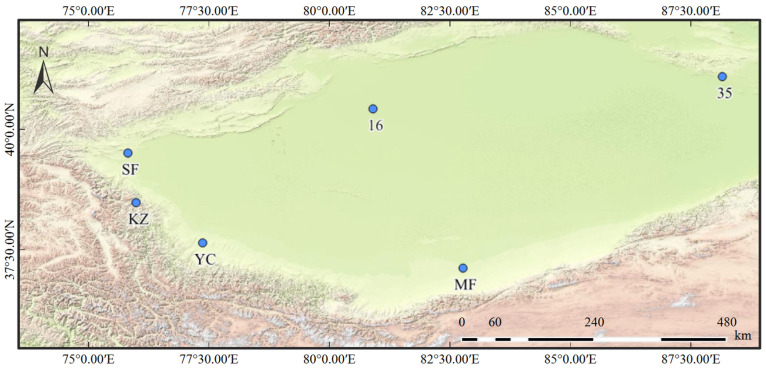
Geographic distribution of sampling sites of *Lycium ruthenicum* in the Tarim Basin, Xinjiang, China. The map presents the spatial locations of sampling sites with distinct longitude, latitude, and topographic characteristics. Labels represent different sampling sites: 35 (Yuli County), MF (Minfeng County), YC (Yecheng County), KZ (Aketao County), SF (Shufu County), and 16 (Awati County). The base map reflects the terrain and geographic features of the Tarim Basin and adjacent areas.

In July 2023, root, fruit, and soil samples of *Lycium ruthenicum* were collected from six sites in the Tarim Basin of China: Awati County (16) in Aksu Prefecture, Aketao County (KZ) in the Kizilsu Kirghiz Autonomous Prefecture, Yecheng County (YC) and Shufu County (SF) in Kashgar Prefecture, Minfeng County (MF) in Hotan Prefecture, and Yuli County (35) in Bayingolin Mongol Autonomous Prefecture (Figure 8). The historical climate data (1993–2023) for each site were extracted from wheatA (https://wheata.cn) using the corresponding latitude and longitude coordinates (Table A1). Drought index (DI) is the ratio of mean evaporation capacity to mean annual precipitation (MAP).

### 4.2. Root, Soil, and Fruit Sample Collection

After removing surface vegetation and coarse debris, we used sterile shovels to gently excavate the soil to extract fine roots (≤2 mm in diameter). The root samples were placed in sterile sampling bags, which were labeled and sealed immediately. The bags were stored in a biological sample container at low temperature and then transported to the laboratory for endophyte isolation and DNA extractions.

Soil samples were simultaneously collected from the 0–20 cm layer using a soil auger (3.5 cm in diameter) in a triangular sampling pattern with three replicates per site.

The three replicates at each sampling site were biological replicates. Specifically, each replicate corresponded to a distinct, healthy black goji individual, i.e., the three replicates at each site were three different black goji plants. Fifty healthy ripening fruits were sampled from each tree, and a total of 150 fruits were collected from each site. The harvested fruits were quickly placed in liquid nitrogen for preservation.

### 4.3. The Determination of Soil Properties

Key soil properties were analyzed using standardized methods [29]: Soil conductivity (SC) was measured using a conductivity meter. Soil pH was determined with a pH meter. Soil organic carbon (SOC) was quantified via potassium dichromate oxidation with external heating. Whereas soil total nitrogen (STN) was analyzed using the semi-micro Kjeldahl method, soil sodium (SNa) was determined via ammonium acetate (NH_4_OAc) extraction followed by flame spectrophotometry (Table A2).

### 4.4. Endophyte Isolation and DNA Extractions

Endophytic bacteria and fungi were isolated from root samples under sterile conditions: healthy roots were rinsed, cut into segments, surface-sterilized sequentially with 75% ethanol and 2% sodium hypochlorite followed by sterile water rinsing (effectiveness verified by culturing the last rinse water), then ground into homogenate; the homogenate was gradiently diluted, spread on NA medium (for bacteria, incubated at 28 ± 2 °C for 24–48 h) and PDA medium (for fungi, added with streptomycin sulfate, incubated at 25 ± 2 °C for 48–72 h), single colonies were purified by streak plate method, and pure cultures were preserved short-term at 4 °C or long-term at −80 °C with glycerol, with all instruments and operations sterilized to avoid contamination.

Endophyte DNA from root samples was extracted using the cetyltrimethylammonium bromide (CTAB) method according to the manufacturer’s instructions. The reagent, which was designed to uncover DNA from trace amounts of sample, has been shown to be effective for the preparation of the DNA of most bacteria. Nuclear-free water was used for the blank. The total DNA was eluted in 50 μL of Elution buffer and stored at −80 °C until measurement in the PCR by LC-Bio Technology Co., Ltd., Hangzhou, Zhejiang Province, China.

### 4.5. PCR Amplification and Sequencing

The V3-V4 region of the bacterial 16S rDNA was amplified using the primers 341F (5′-CCTACGGGNGGCWGCAG-3′) and 805R (5′-GACTACHVGGGTATCTAATCC-3′). The ITS2 region of the fungal sequence was amplified with primers ITS1FI2 (5′-GTGARTCATCGAATCTTTG-3′) and ITS2 (5′-TCCTCCGCTTATTGATATGC-3′). The PCR reaction mixture consisted of 12.5 μL of Phusion Hot Start Flex 2X Master Mix, 2.5 μL of Forward Primer, 2.5 μL of Reverse Primer, 50 ng of template DNA, and ddH_2_O added to a final volume of 25 μL. The PCR amplification protocol was as follows: initial denaturation at 98 °C for 30 s; 35 cycles (32 cycles for ITS2 amplification) of denaturation at 98 °C for 10 s, annealing at 54 °C for 30 s, and extension at 72 °C for 45 s; followed by a final extension at 72 °C for 10 min and holding at 4 °C. The PCR products were purified, diluted, and subjected to paired-end sequencing (2 × 250 bp) on an Illumina NovaSeq 6000 (PE250), provided by LC-Bio Technology Co., Ltd., Hangzhou, China.

### 4.6. Fruit Quality Traits Determination

The fruit anthocyanins were extracted using a 1% acidic ethanol solution (70% ethanol) at a solid-to-liquid ratio of 1:20. The anthocyanin content was then measured using a spectrophotometer (JC-UT2000, Qingdao, China) following established methodology [30]. For proanthocyanidin extraction, a 50% ethanol solution was employed, and its content was determined according to the method described by Li et al. [30]. Total flavonoids were extracted via an organic solvent-based method, and their concentration was quantified using NaNO_2_-Al(NO_3_)_3_-NaOH colorimetry, with rutin as the standard reference. Additionally, fruit polyphenols were extracted through ultrasonic-assisted extraction, and their total content was assessed using the Folin–Ciocalteu (Folin phenol) method.

### 4.7. Data Processing and Analysis

Paired-end sequencing reads were demultiplexed based on barcode information, followed by removal of adapter and barcode sequences. After concatenation and filtering, the DADA2 algorithm in QIIME2 was used for length trimming and denoising to generate amplicon sequence variants (ASVs) [31]. The resulting ASV sequences and abundance table were obtained, and singleton ASVs were removed. Based on the ASV sequences and abundance table, alpha and beta diversity analyses were performed. Alpha diversity was evaluated using three indices: Shannon, Chao1, and Pielou’s evenness. Beta diversity was assessed using four distance metrics—weighted UniFrac, unweighted UniFrac, Jaccard, and Bray–Curtis—followed by Principal Coordinates Analysis (PCoA). For taxonomic annotation, bacterial 16S rDNA ASV sequences were aligned against the SILVA database using the NT-16S reference, and fungal ITS2 ASVs were annotated with the RDP database via the UNITE database. Species abundance in each sample was determined based on the ASV abundance table.

### 4.8. Statistical Analyses

All statistical analyses were performed using IBM SPSS (Version 29; IBM Corp., 2024) and R software (Version 4.2.0; R Core Team, 2022). Pearson correlation analysis was used to evaluate the relationships between root endophytic communities and climate factors, soil properties, or fruit quality traits. One-way analysis of variance (ANOVA) was applied to compare differences in microbial diversity and soil physicochemical properties among different samples. Duncan’s new multiple range test was used for post hoc multiple comparisons. A probability level of *p* < 0.05 was considered statistically significant.

## 5. Conclusions

In this study, we demonstrated that root endophytic bacterial and fungal communities of *Lycium ruthenicum* in the Tarim Basin exhibited significant habitat-specific diversity and distinct structural differentiation. Both bacterial and fungal co-occurrence networks showed high modularity and were primarily shaped by positive synergistic interactions. Keystone microbial taxa were identified as critical drivers in maintaining microbial community stability and function. Root endophytic communities were significantly correlated with climatic conditions, soil properties, and fruit quality traits, suggesting that root endophytes contribute to local environmental adaptation and fruit quality formation in *Lycium ruthenicum*. Notably, this study represents the first systematic investigation revealing the biogeographic distribution patterns of root endophytic microbial communities associated with *Lycium ruthenicum* across the Tarim Basin, as well as their key correlations with fruit quality traits. These findings provide novel insights into the ecological roles of root endophytes in desert medicinal plants and offer a scientific basis for the development and application of beneficial endophytic microbes to enhance fruit yield and quality of *Lycium ruthenicum*.

## Data Availability

The original contributions presented in the study are included in the article. Further inquiries can be directed to the corresponding author.

## Appendix A

**Table A1:** Climatic factors of the sampling sites.

Climate Factors	16	KZ	SF	YC	MF	35
Longitude	80°54′19″	75°59′23″	75°49′12″	77°22′16″	82°46′22″	87°59′13″
Latitude	40°25′12″	38°28′25″	39°30′12″	37°38′17″	37°6′50″	40°55′23″
AL (m)	1046	2107	2000	1865	1420	838
MAP (mm)	73.8	90	72	53.2	36	43
MAT (°C)	11.5	12.6	13.3	14.0	12.1	12.1
WS	4.23	2.81	3.81	3.74	4.22	6.02
DI	28.06	17.09	11.15	54.69	40.36	26.96

AL: altitude; MAP: mean annual precipitation; MAT: mean annual temperature; WS: wind speed; DI: drought index; 16: Awati County; KZ: Aketao County; SF: Shufu County; YC: Yecheng County; MF: Minfeng County; 35: Yuli County.

**Table A2:** The soil properties of sampling sites in the Tarim Basin. Values are means ± SD (*n* = 3 replications).

Sampling Sites	pH	SC (ms/cm)	SOM (g/kg)	STN (mg/kg)	SNa (g/kg)
16	8.65 ± 0.06	10.26 ± 0.91	11.63 ± 1.43	660.99 ± 49.81	9.99 ± 1.35
KZ	8.50 ± 0.26	1.94 ± 1.60	10.37 ± 4.50	530.37 ± 189.63	1.13 ± 0.93
SF	8.65 ± 0.12	3.51 ± 2.35	5.88 ± 2.14	221.69 ± 2.55	2.34 ± 1.72
YC	8.00 ± 0.08	2.43 ± 1.65	9.58 ± 0.78	278.54 ± 60.65	1.83 ± 1.52
MF	8.54 ± 0.15	1.23 ± 0.49	5.89 ± 2.60	263.83 ± 25.29	0.58 ± 0.36
35	8.44 ± 0.12	3.82 ± 0.51	4.83 ± 1.13	114.50 ± 16.58	2.95 ± 0.26

SC: soil conductivity; SOM: soil organic matter; STN: soil total nitrogen; SNa: soil sodium. For sampling site abbreviations, see Table A1.

## References

[1] Huang W., Long C., Lam E. (2018). Roles of plant-associated microbiota in traditional herbal medicine. Trends Plant Sci..

[2] Wu W., Chen W., Liu S., Wu J., Zhu Y., Qin L., Zhu B. (2021). Beneficial relationships between endophytic bacteria and medicinal plants. Front. Plant Sci..

[3] Semenzato G., Fani R. (2024). Endophytic bacteria: A sustainable strategy for enhancing medicinal plant cultivation and preserving microbial diversity. Front. Microbiol..

[4] Zhang Y.Y., Wen L., Wang T.T., Li Y.Z. (2025). Role of endophyte in salinity stress amelioration by growth, physiology, and biochemistry mechanisms of defense: A meta-analysis. Physiol. Plant..

[5] Wang Y., Chen N., Deng K., Zhong X., Li Z., Li L., Xu D. (2025). Effects and molecular mechanism of endophytic elicitors on the accumulation of secondary metabolites in medicinal plants. Front. Microbiol..

[6] Lv J., Yang S., Zhou W., Liu Z., Tan J., Wei M. (2024). Microbial regulation of plant secondary metabolites: Impact, mechanisms and prospects. Microbiol. Res..

[7] Liu J., Abdelfattah A., Norelli J., Burchard E., Schena L., Droby S., Wisniewski M. (2018). Apple endophytic microbiota of different rootstock/scion combinations suggests a genotype-specific influence. Microbiome.

[8] Nerva L., Sandrini M., Moffa L., Velasco R., Balestrini R., Chitarra W. (2022). Breeding toward improved ecological plant-microbiome interactions. Trends Plant Sci..

[9] Su J., Wang Y., Bai M., Peng T., Li H., Xu H.J., Guo G., Bai H., Rong N., Sahu S.K. (2023). Soil conditions and the plant microbiome boost the accumulation of monoterpenes in the fruit of *Citrus reticulata* ‘Chachi’. Microbiome.

[10] Han X., Zhang K.P., Zhang Z.A., Dai C.C., Chen F. (2025). Endophytes drive the biosynthesis and accumulation of sesquiterpenoids in *Atractylodes lancea* (Thunb.) DC. Ind. Crops Prod..

[11] Li J., Liu H., Wang J., Macdonald C.A., Singh P., Cong V.T., Klein M., Delgado-Baquerizo M., Singh B.K. (2025). Drought-induced plant microbiome and metabolic enrichments improve drought resistance. Cell Host Microbe.

[12] Yu J.B., Bai M., Wang C.Y., Wu H., Liang X.X. (2024). Regulation of secondary metabolites accumulation in medicinal plants by rhizospheric and endophytic microorganisms. Microbiol. Res..

[13] Zareb A., Banachewicz P., Havrysh P., Błaszczyk L., Hammad T., Meftah C., Salamon S. (2025). Endophytic fungal communities of *Calicotome spinosa*—An important medicinal plant of Tizi-Ouzou (Algeria). J. Appl. Genet..

[14] Wang C.C., Ma S.M., Sun F.F., Wei B., Nie Y.B. (2021). Spatial genetic patterns of the medicinal and edible shrub Lycium ruthenicum (Solanaceae) in arid Xinjiang, China. Tree Genet. Genomes.

[15] Wang H., Li J., Tao W., Zhang X., Gao X., Yong J., Zhao J., Zhang L., Li Y., Duan J.A. (2018). Lycium ruthenicum studies: Molecular biology, Phytochemistry and pharmacology. Food Chem..

[16] Xin W., Zhang J., Yu Y., Tian Y., Li H., Chen X., Li W., Liu Y., Lu T., He B. (2024). Root microbiota of tea plants regulate nitrogen homeostasis and theanine synthesis to influence tea quality. Curr. Biol..

[17] Liu Q., Hu D., Qiao Y., Zai X., Hao X., Zong Y., Zhang D., Shi X., Zhang F., Li P. (2025). Phyllosphere microbes in foxtail millet primarily affect quality by modulating coloration and bitter compounds. Microbiome.

[18] Dubey A., Malla M.A., Kumar A., Dayanandan S., Khan M.L. (2020). Plants endophytes: Unveiling hidden agenda for bioprospecting toward sustainable agriculture. Crit. Rev. Biotechnol..

[19] Godara H., Ramakrishna W. (2023). Endophytes as nature’s gift to plants to combat abiotic stresses. Lett. Appl. Microbiol..

[20] Kumar V., Nautiyal C.S. (2022). Plant abiotic and biotic stress alleviation: From an endophytic microbial perspective. Curr. Microbiol..

[21] Bokhari A., Essack M., Lafi F.F., Andres-Barrao C., Jalal R., Alamoudi S., Razali R., Alzubaidy H., Shah K.H., Siddique S. (2019). Bioprospecting desert plant Bacillus endophytic strains for their potential to enhance plant stress tolerance. Sci. Rep..

[22] Sharma M., Sood G., Chauhan A. (2025). Bacterial endophytes of medicinal plants: Applications and recent developments. Curr. Microbiol..

[23] Rutkowska N., Daroch M., Marchut-Mikołajczyk O. (2025). Exploring the diversity and genomics of cultivable Bacillus-related endophytic bacteria from the medicinal plant *Galium aparine* L.. Front. Microbiol..

[24] Tang M., Yao X.Y., Xu J.Y., Wu J.Y., Sun K., Dai C.C., Chen F. (2025). Auxin signaling activated by endophytic Cross-Kingdom synthetic microbiota improves the quality of *Atractylodes chinensis*. Microbiol. Res..

[25] Sun K., Pan Y.T., Jiang H.J., Xu J.Y., Ma C.Y., Zhou J., Liu Y., Shabala S., Zhang W., Dai C.C. (2024). Root endophyte-mediated alteration in plant H_2_O_2_ homeostasis regulates symbiosis outcome and reshapes the rhizosphere microbiota. J. Exp. Bot..

[26] Gostinčar C., Stajich J.E., Gunde-Cimerman N. (2023). Extremophilic and extremotolerant fungi. Curr. Biol..

[27] Liu H., Li J., Singh B.K. (2024). Harnessing co-evolutionary interactions between plants and Streptomyces to combat drought stress. Nat. Plants.

[28] Li N., Li G., Huang X., Ma L., Wang D., Luo Y., Cao X., Zhu Y., Mu J., An R. (2026). Large-scale multi-omics unveils host-microbiome interactions driving root development and nitrogen acquisition. Nat. Plants.

[29] Page A.L. (1982). Methods of Soil Analysis: Part 2. Chemical and Microbiological Properties.

[30] Li G., Qin B., Li S., Yin Y., Zhao J., An W., Cao Y., Mu Z. (2020). LbNR-derived nitric oxide delays Lycium fruit coloration by transcriptionally modifying flavonoid biosynthetic pathway. Front. Plant Sci..

[31] Bolyen E., Rideout J.R., Dillon M.R., Bokulich N.A., Abnet C.C., Al-Ghalith G.A., Alexander H., Alm E.J., Arumugam M., Asnicar F. (2019). Reproducible, interactive, scalable and extensible microbiome data science using QIIME 2. Nat. Biotechnol..

